# Knockdown of TPI in human dermal microvascular endothelial cells and its impact on angiogenesis *in vitro*

**DOI:** 10.1371/journal.pone.0294933

**Published:** 2023-12-20

**Authors:** Christina Herre, Arpenik Nshdejan, Robert Klopfleisch, Giuliano Mario Corte, Mahtab Bahramsoltani

**Affiliations:** 1 Institute of Veterinary Anatomy, School of Veterinary Medicine, Freie Universität Berlin, Berlin, Germany; 2 Institute of Veterinary Pathology, School of Veterinary Medicine, Freie Universität Berlin, Berlin, Germany; 3 Department of Veterinary Medicine, Institute of Veterinary Anatomy, Universität Zürich, Zurich, Switzerland; Duke University School of Medicine, UNITED STATES

## Abstract

**Introduction:**

Angiogenic behaviour has been shown as highly versatile among Endothelial cells (ECs) causing problems of *in vitro* assays of angiogenesis considering their reproducibility. It is indispensable to investigate influencing factors of the angiogenic potency of ECs.

**Objective:**

The present study aimed to analyse the impact of knocking down triosephosphate isomerase (TPI) on *in vitro* angiogenesis and simultaneously on vimentin (VIM) and adenosylmethionine synthetase isoform type 2 (MAT2A) expression. Furthermore, native expression profiles of TPI, VIM and MAT2A in the course of angiogenesis *in vitro* were examined.

**Methods:**

Two batches of human dermal microvascular ECs were cultivated over 50 days and stimulated to undergo angiogenesis. A shRNA-mediated knockdown of TPI was performed. During cultivation, time-dependant morphological changes were detected and applied for EC-staging as prerequisite for quantifying *in vitro* angiogenesis. Additionally, mRNA and protein levels of all proteins were monitored.

**Results:**

Opposed to native cells, knockdown cells were not able to enter late stages of angiogenesis and primarily displayed a downregulation of VIM and an uprise in MAT2A expression. Native cells increased their TPI expression and decreased their VIM expression during the course of angiogenesis *in vitro*. For MAT2A, highest expression was observed to be in the beginning and at the end of angiogenesis.

**Conclusion:**

Knocking down TPI provoked expressional changes in VIM and MAT2A and a deceleration of *in vitro* angiogenesis, indicating that TPI represents an angiogenic protein. Native expression profiles lead to the assumption of VIM being predominantly relevant in beginning stages, MAT2A in beginning and late stages and TPI during the whole course of angiogenesis *in vitro*.

## Introduction

The process of building new blood vessels due to endothelial sprouting or intussusceptive growth, is defined as angiogenesis [1]. Sprouting angiogenesis *in vivo* is based on the specialization of endothelial cells (ECs) into tip cells, stalk cells and phalanx cells. An angiogenic stimulus, e.g. vascular endothelial growth factor A (VEGF-A), induces tip cell differentiation and filopodia formation via the vascular endothelial growth factor receptor-2 (VEGFR-2). While tip cells migrate towards the stimulus, stalk cells differentiate and proliferate in order to elongate the sprout. Guidance for the sprout growth is mainly conducted by stalk cells expressing predominantly vascular endothelial growth factor receptor-1 (VEGFR-1). After lumenogenesis, phalanx cells promote vessel integrity and stabilization [2,3].

Respectively, an excessive or deficient course of angiogenesis promotes many pathological events, such as tumor growth or dysfunctional tissue repair. Currently, the research field of angiogenesis is mainly focusing on cancer treatment, tissue engineering and wound healing [4,5]. In practice, *in vitro* models are frequently used in order to reduce time and cost, be carried out expeditiously, and mainly to reduce animal experiments in the sense of the 3R principle. Nevertheless, *in vitro* models display inconsistencies regarding their reproducibility, based on the inhomogeneous use of models and the heterogeneous character of ECs [2,6–9].

Variations considering the angiogenic potency of ECs were also shown by Bahramsoltani et al. [10–13]. Several batches of capillary-derived primary cell cultures of human microvascular ECs were cultivated *in vitro* while using a newly established all-in-one assay, which comprises all phases of angiogenesis. Partially, cells were not able to enter all defined stages of angiogenesis *in vitro*, hence being classified as non-angiogenic ECs. Comparatively, angiogenic ECs ran through each angiogenic stage *in vitro* chronologically. By searching for proteomic differences between both batches of ECs, seven proteins were detected solely in angiogenic ECs and one protein in non-angiogenic ECs [14]. Three of these proteins were triosephosphate isomerase (TPI), vimentin (VIM) and S–adenosylmethionine synthetase isoform type 2 (MAT2A) [15].

TPI was one of the proteins found in angiogenic ECs [14]. It is a dimeric, non-allosteric enzyme which is primarily known for its catalytic activity in glycolytic pathways. Hereby, it facilitates the interconversion of dihydroxyacetone phosphate and D–glyceraldehyde–3–phosphate [16,17]. Recently, several additional functions were attributed to TPI which do not necessarily involve catalysis, defining TPI as a moonlighting protein [17–19]. Considering ECs, an increase in TPI expression induced by hypoxia had been demonstrated in capillary ECs [20]. Besides hypoxia, it was also shown that glycolysis can be stimulated via VEGF in ECs *in vitro* [21]. Furthermore, TPI expression and glycolic metabolism appeared to be higher in angiogenic ECs using the generated energy for cell motility and proliferation [15,22].

VIM represents an additional protein being detected in angiogenic ECs [14]. As a type III intermediate filament protein, VIM is mostly known for stabilizing intracellular structures, influencing cell shape and contractility. Besides intracellular signalling pathways, VIM is also highly involved in extracellular regulations affecting divers physiological and pathological events, such as cell growth and differentiation, wound healing and viral infections [23–25]. By knocking down VIM, it had been shown that this protein is essential for ECs to run through all stages of angiogenesis *in vitro*. Over the course of angiogenesis *in vitro*, highest VIM expressions were detected in the beginning stages, indicating its involvement in cell migration [15,24,26]. Additionally, dynamics in VIM expression were detected in microvascular ECs, adjusting cell adhesion and motility to environmental stress [27,28]. Up until today, VIM is being analysed to reveal further molecular mechanisms that are involved in the process of angiogenesis [29].

MAT2A was the protein found in non-angiogenic ECs [14]. In most tissues, this enzyme is mainly encoded by the MAT2A-gene. Its primary function is catalysing the synthesis of S-adenosylmethionine (SAM) from methionine and adenosine triphosphate (ATP) [30–32]. SAM represents a product of the methionine cycle and thereby is involved in synthesizing polyamines, homocysteine and reduced glutathione. By being a major methyl-donor, it is highly involved in methylation reactions, e.g. protein- and DNA-methylation. Hence, it regulates cellular metabolism on genetic and molecular levels [33–35]. In ECs, it was shown that an inhibition of methylation led to an increase in VEGF-A expression followed by the differentiation of endothelial cells [36]. Moreover, a hypermethylation by supplying SAM, ECs were hindered to migrate and proliferate [37]. Currently, there is hardly any information about MAT2A and its role in angiogenesis.

This present study is based on the hypothesis of TPI being an essential angiogenic protein for angiogenesis *in vitro*. The aim of this study was to detect morphological and molecular changes in human dermal microvascular endothelial cells (HDMEC) running through *in vitro* angiogenesis after knocking down TPI. Additionally, native expression of TPI, VIM and MAT2A and expressional changes of VIM and MAT2A expression in knockdown cells were analysed.

## Cells, materials and methods

### Plasmids, primers and shRNA

Design and synthesis of short hairpin RNA targeting TPI-mRNA (shTPI) was executed according to previous studies [15,38,39]. In brief, four genetic shTPI sequences were generated being structured sense-loop-antisense (loop sequence TTCAAGAGA). First, knockdown effectiveness and the power of each hairpin construct were analyzed in HEK 293T cells *in vitro* by infecting cells with each hairpin construct individually and including a cellular induction of TPI overexpression simultaneously (TPI^+^-forward GCGGGATCCGCCACCATGGCGGAGGACGGCGAG, TPI^+^-reverse GCGGATATCTCGTTGTTTGGCATTGATGATGTCC). The overexpressed TPI was tagged with V5 epitope, Western Blot analysis using Rabbit polyclonal Anti-V5 tag primary antibodies (Abcam, Cambridge, UK, ab15828, 1:5,000) and donkey Anti-Rabbit IgG HRP Linked species specific F(ab’)2 fragmentsecondary antibodies (GE Healthcare, Freiburg, Germany, NA9340, 1:10,000) revealed the specific construct displaying the highest knockdown efficiency (shTPI target sequence GCTGAAGTCCAACGTCTCTGA). Based on the shTPI sequence, a nontargeting sequence was designed consisting of the identical amount and type of nucleotides serving as control (shSCR target sequence GCGCAGTGCCCGTACATATTA). After attaching an U6 promotor cassette to pFUGW plasmid, containing the DNA fragments encoding the hairpins, it was used as lentiviral expression vector, additionally containing the genetic information for enhanced green fluorescent protein (eGFP). The viral particles displayed a titer in the range of 0.7–0.9 x10^6^ IU/μl and were used in 20-fold concentration. The amount of virus was determined after the initiation and analysis of trial runs using virus in a 10-, 20- and 30-fold concentration individually.

### Cells, media and cultivation

Human dermal microvascular endothelial cells (HDMECs) were purchased from LONZA Bioscience (Basel, Switzerland, HMVEC–dBl–Neo, Cat. No. CC–2813). Distributor’s analysis of CD31/105, von Willebrand Factor VIII and positive uptake for acetylated low density lipoprotein guaranteed EC population. In total, two batches (HD1 and HD2) were acquired and cultivated in EBM^TM^–2 Endothelial Cell Growth Basal Medium–2 (LONZA, Basel, Switzerland, Cat. No. CC–00190860) as basal medium (BM). EGM^TM^–2 MV Microvascular Endothelial SingleQuots^TM^ Kit (LONZA, Basel, Switzerland, Cat. No. CC–4147), containing Fetal Bovine Serum, growth factors, antioxidants, antibiotics, antimycotics and anti-inflammatories, was added to the BM in order to stimulate the angiogenic response in HD1 and HD2. The detailed composition of media was according to the previously described study [15]. Exchange of media were executed twice a week.

### *In vitro* angiogenesis assay

For cultivation, 24-well-culture plates (Corning Life Sciences, Amsterdam, Netherlands, Cat. No. 3738) were used. Each well was covered with 0,5ml gelatine (Sigma Aldrich, St. Louis, MO, USA, Cat. No. G6144, 1,5% in PBS) and incubated for 20 minutes at 37°C. Per well, 4.5 x 10^4^ cells of both batches were seeded in third passage and cultivated up to 50 days at 37°C in a 5% CO_2_ humidified atmosphere (INCO2/1, Memmert GmbH & Co. KG, Schwabach, Germany). On day one, in both batches respectively, a third of cells either got infected with viral particles owning shTPI and initiating the knockdown (sh_1_, sh_2_), or with lentiviruses consisting shSCR serving as control group (SCR_1_, SCR_2_), or they remained unmodified (N_1_, N_2_). Twice a week, digital pictures were taken of four visual fields of each well using an inverted microscope (LEICA DMi8; Leica Microsystems, Wetzlar, Germany), LEICA MC170 HD video camera (Leica Microsystems, Wetzlar, Germany) and the imaging and analysis software Leica Application Suite X (LAS X Version 3.4.2, Leica Microsystems, Wetzlar, Germany). According to the all-in-one angiogenesis assay [10–13,40], the morphology of ECs in the micrographs were analysed and assigned to the respective stage of angiogenesis *in vitro* (Table 1. [12]). For quantifying angiogenesis, the sum of the allocated stages of each visual field over the time was computed separately for each group of HD1 and HD2 (S^group^). Further, the arithmetic mean of all the sums of each group was calculated and compared ($\bar{S^{\mathrm{group}}}$).

**Table 1:** Definition of stages of angiogenesis *in vitro* and description of cell morphology within the different stages [12].

Stage no.	Morphology of endothelial cells
**Stage 1**	Confluent monolayer Polygonal shaped cells
**Stage 2**	Endothelial sprouting, late phase >50% elongated shaped cells
**Stage 3**	Linear side–by–side arrangement, late phase >50% linearly arranged cells
**Stage 4**	Networking Network of linearly arranged cells
**Stage 5**	Three-dimensional organisation, early phase Appearance of capillary–like structures (linear structures of endothelial cells with a diameter of more than 28 μm; for these structures an internal lumen was shown by electron microscopy)
**Stage 6**	Three–dimensional organisation, late phase All linearly arranged cells form capillary–like structures; dissolution of cell layer on the bottom

### Quantitative analysis of VIM, TPI, MAT2A transcripts via RT–qPCR

At day 5, 15, 25 and 50, harvesting of cells of each group was carried out using Hydroxyethylpiperazine Ethane Sulfonic acid, Trypsin/EDTA and Trypsin Neutralisation Solution (LONZA, Basel, Switzerland, ReagentPackTM Subculture Reagents, Cat. No. CC–5034). After centrifugation, cell pellets were deeply frozen in liquid nitrogen and stored at -76°C. RNA isolation and digestion of remaining DNA was executed using Total RNA Kit, peqGold (Peqlab/VWR, Darmstadt, Germany, Cat. No. 12–6834) and TURBO^TM^ DNase (ThermoFisher Scientific, Bremen, Germany, Cat. No. AM2238). Applying SuperScript III Reverse Transcriptase (ThermoFisher Scientific, Bremen, Germany, Cat. No. 18091050), RNA was reverse transcribed for cDNA synthesis. Quantitative PCR was performed with triplicates of all samples, utilising Maxima SYBR Green qPCR Master Mix (2x) (ThermoFisher Scientific, Bremen, Germany, Cat. No K0223), Rotor–Gene 6000 (Qiagen, Hilden, Germany) and Rotor–Gene Q 2.3.5 software. According to the previously published article, GAPDH was shown to be the most stable reference gene and was used as normalizer gene[15]. For every gene, the respective standard curve displayed the calibrator sample and the amplification efficiency. The C_t_ difference between gene of interest and calibrator was determined and adjusted to the amplification efficacy. Finally, samples were normalized to GPDH. All primers are listed in the corresponding article [15].

### Western blot analysis

The method of protein detection, chemicals and antibodies were applied as previously described [15]. In brief, 20μg protein per sample was deployed in triplicates and separated by 12% Bis-Tris SDS-PAGE sodium dodecyl sulfate–polyacrylamide gel electrophoresis. Proteins were transferred onto nitrocellulose membranes by electroblotting. As primary antibodies, VIM (DAKO, Hamburg, Germany, M7020, 1:500), TPI (Santa Cruz, Heidelberg, Germany, H–11, 1:200) and MAT2A (Santa Cruz, Heidelberg, Germany, B–10, 1:200) were used. Additionally, Actin (Novus Biologicals, Centennial, CO, USA, AC–15, 1:5,000) served as the internal control. For VIM and actin (ACT) detection, a further incubation in sheep anti–mouse IgG secondary antibody (GE Healthcare, Freiburg, Germany, NA9310, 1:5,000) was performed. SignalFireTM ECL Reagent (Cell Signal technology, Frankfurt, Germany, Cat No. 6883) was used for visualization. Densitometric raw volume of all samples were determined by GeneTools software version 4.03.05.0 (SynGene, Cambridge, England). Signal intensity of all values was normalised to the respective Actin.

### Statistics

Statistical examination of data was performed using SPSS Statistics (SPSS Statistics 29, IBM Corporation, Armonk, NY, USA). First, the Shapiro-Wilk test was carried out revealing value distributions. Normally distributed data is presented as mean ± standard deviation, non-normally distributed as median ± standard error. By executing Student’s *t* test for unpaired data or Mann–Whitney U test, two independent groups were compared. For multiple groups, analysis was done using one-way ANOVA or Kruskal-Wallis test followed by post hoc Dunn-Bonferroni test, respectively. *P*-value of 0.05 or less were defined as statistically significant.

## Results

### *In vitro* angiogenesis of N_1_ and N_2_, SCR_1_ and SCR_2_

**Fig 1:**
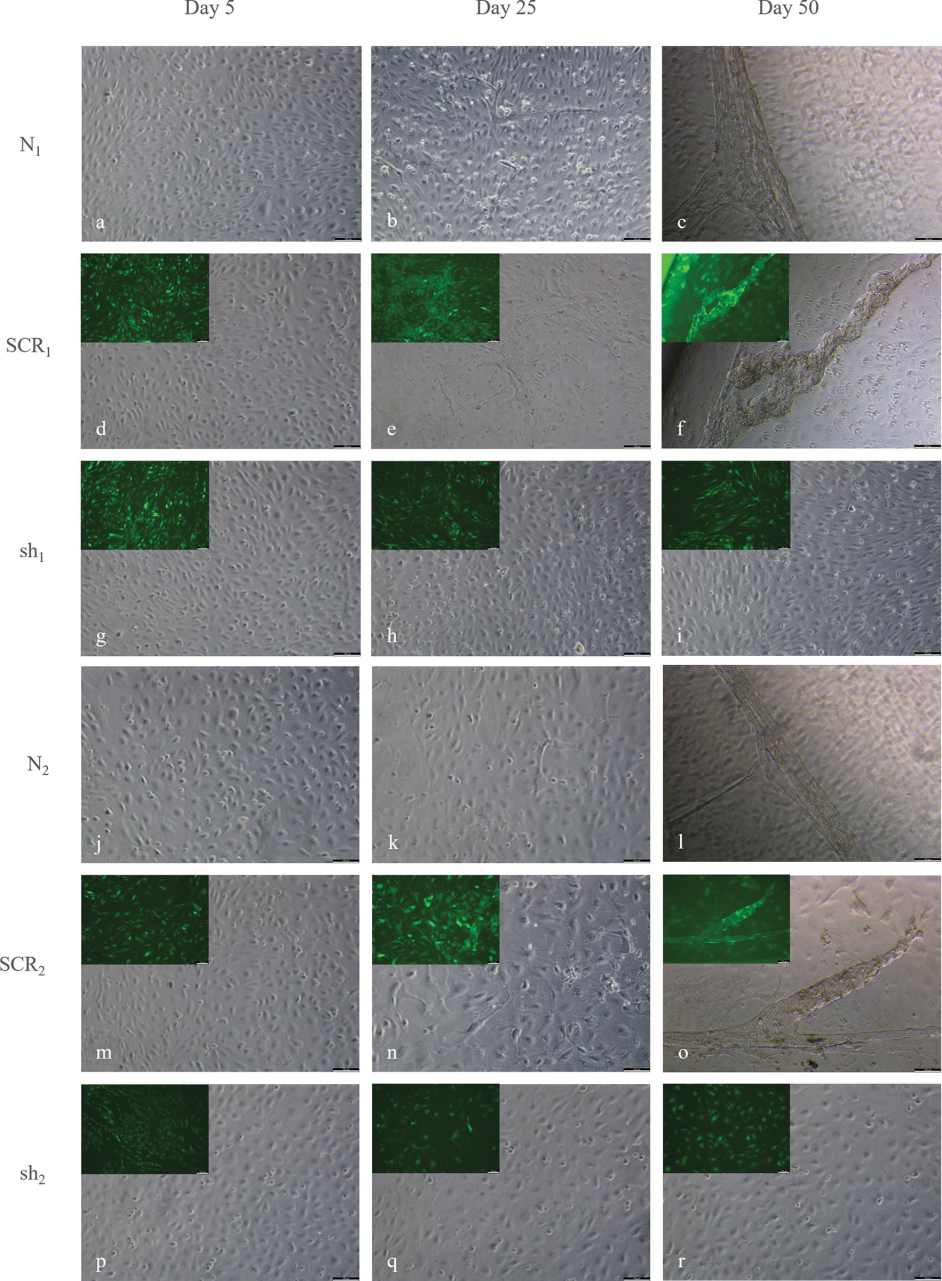
Morphological changes of ECs during angiogenesis *in vitro*. Native (a, b, c), control (d, e, f) and knockdown cells (g, h, i) of HD1 are presented at day 5 (a, d, g), 25 (b, e, h) and 50 (c, f, i)., followed by native (j, k, l), control (m, n, o) and knockdown cells (p, q, r) of HD2 at day 5 (j, m, p), 25 (k, n, q), and 50 (l, o, r). In the upper left corner of sh and SCR micrographs, GFP control is shown. In all groups, cells were polygonal and elongated shaped at day 5, representing stage 1–2 (a, d, g, j, m, p). At day 25, native and control cells of both batches displayed networking structures of stage 4–5 (b, e, k, n), followed by three-dimensional organisation of stage 6 (c, f, l, o). In contrast, knockdown cells remain in stage 3, showing linear side-by-side arrangements as the furthest stage of differentiation (h, i, q, r). Scale bars = 100 μm.

Native cells of both batches were able to undergo the angiogenic cascade chronologically. From the beginning of cultivation, cell population of N_1_ displayed a higher cell density than N_2_. In N_1_, endothelial sprouting was already visible at day 5, by polygonal shaped cells starting to elongate (Fig 1A). From day 25 onwards, cells displayed an early phase of three-dimensional organisation, representing stage 4 to 5 (Fig 1B). Followed by the dissolution of capillary-like structures from the bottom of the cell culture plates of stage 6, which was observed from day 39 (Fig 1C). In N_2_, cells demonstrated sprouting activity from day 5 onwards (Fig 1J). In general, sprouting activity was less visible in cells of HD2 than in HD1. From day 15, linearly arranged cells generated networks (Fig 1K) and ended up building capillary-like structures after 43 days of cultivation (Fig 1L). Median and standard error of sums of assigned stages of angiogenesis (S) were calculated and compared using the Kruskal-Wallis test, followed by the post hoc Dunn-Bonferroni test. Resulting in $\bar{S^{N_{1}}}$ = 53.4 ± 2.7 for N_1_ and $\bar{S^{N_{2}}}$ = 51.5 ± 3.4 for N_2_ with N_2_ being significantly smaller than N_1_ (*p*<0.001).

Similar to N_1_ and N_2_, cells of SCR_1_ and SCR_2_ displayed a high amount of elongated shaped cells at day 5 (Fig 1D and 1M). Already at day 22, ECs of both batches reached stage 5 by networking and starting a three-dimensional organisation (Fig 1E and 1N). Finally, SCR_1_ entered stage 6 after 32 days (Fig 1F) and SCR_2_ after 39 days (Fig 1O). By eGFP serving as an infection control, the fluorescent signal was surveyed at each detection day. For control groups of both batches, a consistent infection was visible (Fig 1D–1F and 1M–1O). For $\bar{S^{{SCR}_{1}}}$ a value of 55.5 ± 3.0 was determined, being significantly higher than $\bar{S^{{SCR}_{2}}}$ = 52.1 ± 5.1 (*p*<0.001). No differences were detectible between native and control groups of both batches.

### *In vitro* angiogenesis of sh_1_ and sh_2_

In the beginning of cultivation, no differences in sh_1_ and sh_2_ in comparison to native and control cells were visible considering their morphology. At day 5, cells were assigned to stages 1 and 2 (Fig 1G and 1P). However, a delay in entering next stages was visible in the following days. While native and control groups of both batches already entered stage 3 at day 8, sh_1_ was able to build linear side-by-side arrangements at day 11 and sh_2_ at day 15 (Fig 1H and 1Q). Stage 3 represents the furthest stage knockdown cells were able to enter during the cultivation period of 50 days. For all groups of both batches, mean values and standard deviations of all assigned stages are shown in S1 Table. The course of *in vitro* angiogenesis of respective groups are visualized in S1 Fig. A small number of cells of sh_1_ started to enter stage 4 from day 18 onwards ending up in stage 5 at day 50. For these cells no fluorescence was observed, which led to the exclusion of further morphological analysis. Otherwise, eGFP signal was persistent throughout the whole cultivation in sh_1_ and sh_2_ (Fig 1G–1I and 1P–1R). For sh_1_, a sum of $\bar{S^{{sh}_{1}}}$ = 44.5 ± 3.1 was calculated, thus a significant lower value than $\bar{S^{N_{1}}}$ and $\bar{S^{{SCR}_{1}}}$ (*p*<0.001). Alike, sums of N_2_ and SCR_2_ were significantly higher than sh_2_, resulting in $\bar{S^{{sh}_{2}}}$ = 37.7 ± 3.4 (*p*<0.001). Comparing sums of knockdown groups in between batches, sh_1_ showed a significantly higher value than sh_2_ (*p*<0.001).

### TPI, VIM and MAT2A expression in N_1_ and N_2_

**Fig 2:**
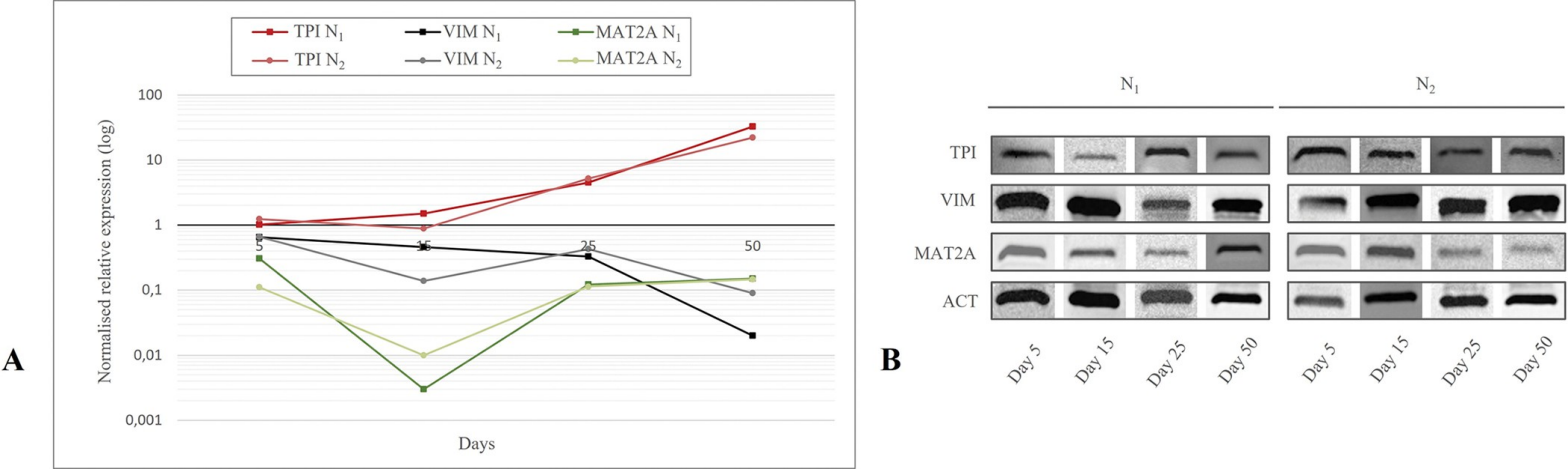
Expression of TPI, VIM and MAT2A in native cells. **A.** Changes of TPI, VIM and MAT2A mRNA expression of N_1_ and N_2_ in the course of angiogenesis *in vitro*. Predominantly increasing expression of TPI, decreasing expression of VIM and falling and rising expression of MAT2A was statistically analysed using Kruskal-Wallis test and post hoc Dunn-Bonferroni test (*p*<0.05). **B.** Western blot analysis of TPI, VIM and MAT2A in N_1_ and N_2_ at day 5, 15, 25 and 50 using ACT as internal control.

At day 5, 15, 25 and 50 of cultivation, mRNA and protein expression of TPI, VIM and MAT2A were examined for native cells of both batches. In N_1_, TPI mRNA expression was stable at first, followed by a significant increase at day 25 (*p*<0.01) and day 50 (*p*<0.001). Whereas in N_2_, TPI mRNA expression decreased at day 15 (*p*<0.05) and increased on following detection days (*p*<0.001) emerging in a significantly lower expression of TPI in N_2_ at day 15 compared to N_1_ (*p*<0.05, Fig 2A). On protein level, TPI was detectible during the whole angiogenic cascade *in vitro* (Fig 2B). While N_1_ displayed a significant decline of VIM mRNA between day 5 and 50 (*p*<0.05), N_2_ VIM expression fluctuated starting with a decrease (*p*<0.001) and an increase (*p*<0.001), followed by a down scale (*p*<0.001). At day 15, VIM mRNA expression was higher in N_1_ than N_2_ (p<0.05), whereas VIM mRNA was significantly lower in N_1_ at day 50 compared to N_2_ (*p*<0.001, Fig 2A). Western blot analysis showed stable protein levels of VIM (Fig 2B). Considering MAT2A, both native cell groups decreased their mRNA expression at day 15 (*p*<0.01), followed by an uprise at day 25 (*p*<0.05). Solely at day 5, MAT2A expression was observed to be significantly higher in N_1_ than in N_2_ (*p*<0.01, Fig 2A). Western Blot analysis displayed bright protein bands and lower protein values for N_1_ at day 25 (*p*<0.05) and for N_2_ at day 25 and 50 (*p*<0.05, Fig 2B, S3 Table).

### Expression of TPI, VIM and MAT2A in sh_1_ and sh_2_

**Fig 3:**
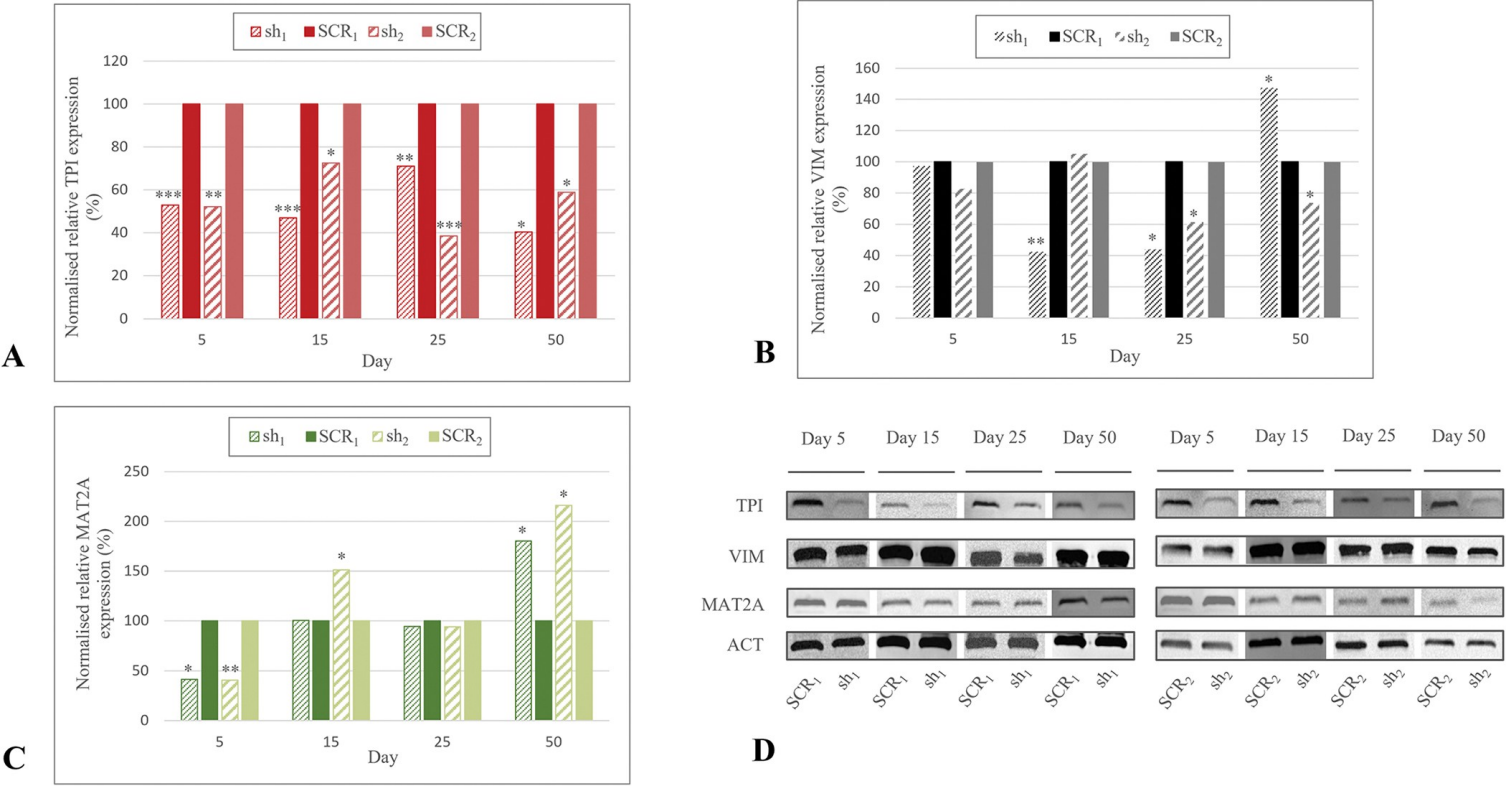
Expression of TPI, VIM and MAT2A in sh_1_ and sh_2_. Statistical analysis was performed using the Mann-Whitney U test for unpaired data. **A.** Normalised relative TPI expression of knockdown and control groups of HD1 and HD2. At each detection day, TPI expression was downregulated in sh_1_ and sh_2_. **B.** Normalised relative VIM expression of sh_1_, SCR_1_, sh_2_ and SCR_2_. VIM mRNA was significantly downregulated in sh_1_ at day 15 and 25 and in sh_2_ at day 25 and 50. Significant uprise in expression was observed in sh_1_ at day 50. **C.** Normalised relative MAT2A expression of sh_1_, SCR_1_, sh_2_ and SCR_2_. MAT2A displayed a decrease in sh_1_ and in sh_2_ at day 5. Significantly higher expression was observed in sh_1_ at day 15 and 50 and in sh_2_ at day 50. **D.** Western blot analysis of TPI, VIM and MAT2A in knockdown and control groups of HD1 and HD2 at day 5, 15, 25 and 50. ACT was used as an internal control. *p<0.05, **p<0.01, ***p<0.001.

For knockdown control, eGFP was detected and TPI expression was analysed via RT-qPCR and Western blot. TPI mRNA was significantly downregulated in sh_1_ compared to SCR_1_ at day 5 and 15 (*p*<0.001), 25 (*p*<0.01) and 50 (*p*<0.05). Coincidentally, TPI mRNA expression in sh_2_ was significantly lower than in SCR_2_ at day 5 (*p*<0.01), 15 (*p*<0.05), 25 (*p*<0.001) and 50 (*p*<0.05, Fig 3A). For sh_1_ and sh_2_, protein expression of TPI was decreased in comparison to control groups, respectively (Fig 3D). By comparing knockdown groups of both batches, TPI mRNA expression was significantly higher in sh_1_ than in sh_2_ (*p*<0.01).

For assessing expressional changes in VIM and MAT2A induced by knocking down TPI, knockdown groups were compared to their respective control groups. VIM mRNA was downregulated in sh_1_ at day 15 (*p*<0.01) and 25 (*p*<0.05). In contrast, an increase of mRNA was observed at day 50 (*p*<0.05). In sh_2_, VIM mRNA expression was significantly downregulated at day 25 and day 50 (*p*<0.05, Fig 3B). No difference in protein expression was detected in between knockdown and control groups at any day (Fig 3D). At day 5, VIM mRNA was expressed less in sh_1_ than in sh_2_ (*p*<0.01), whereas sh_1_ displayed a higher VIM mRNA expression at day 50 (*p*<0.001).

For MAT2A, mRNA expression in sh_1_ was less than SCR_1_ at day 5 (*p*<0.05) and higher at day 50 (*p*<0.05). In sh_2_, MAT2A expression was decreased at day 5 (*p*<0.01) and upregulated at day 15 (*p*<0.05) and 50 (*p*<0.05, Fig 3C). On protein level, MAT2A was detectable at each day of investigation in both batches (Fig 3D). At day 50, sh_1_ and SCR_1_ displayed stronger protein bands and higher values (*p*<0.01), whereas sh_2_ and SCR_2_ presented brighter protein bands and lower values (*p*<0.001, *p*<0.05, S3 Table). For MAT2A mRNA, no differences in between sh_1_ and sh_2_ were exposed. Median and standard error of mRNA expression of all three proteins is provided in S2 Table. Furthermore, S3 Table displays median and standard error of respective protein expressions.

## Discussion

ECs display diversity considering their angiogenic behaviour while running through angiogenesis *in vitro* causing a lack of reliability of *in vitro* models [2,6–13]. Influencing factors on angiogenic potency of ECs must get investigated. This study is mainly focusing on the enzyme TPI and its impact on HDMECs running through angiogenesis *in vitro*. After knocking down TPI, morphological and molecular changes of VIM and MAT2A expression were examined. Additionally, native expression of TPI, VIM and MAT2A were determined during the course of angiogenesis *in vitro*.

As previously published, HD1 and HD2 are characterized as angiogenic ECs, being able to run through the whole angiogenic cascade *in vitro*. Additionally, analysis of VEGFR-1 and VEGFR-2 expression in both batches indicated a higher amount of stalk cells in the cell population of HD1 than in HD2 [15]. Endothelial stalk cells are highly proliferative in order to elongate the sprout during angiogenesis [2,3,41]. In this study, the strong proliferative character of cells of HD1 was visible by a persistently higher cell density in all groups of HD1 compared to HD2. In addition, more cells were able to enter late stages of angiogenesis, resulting in significant higher values of $\bar{S^{N_{1}}},\bar{S^{{SCR}_{1}}}$ and $\bar{S^{{sh}_{1}}}$ than $\bar{S^{N_{2}}},\bar{S^{{SCR}_{2}}}$ and $\bar{S^{{sh}_{2}}}$, respectively. Further, knockdown cells of HD1 were able to generate GFP-negative and therefore non-infected cells which were able to enter the angiogenic cascade and precede to further stages. In contrast, being less proliferative, sh_2_ was not able to compensate manipulation and remained in early stages.

Considering native mRNA expression of TPI during angiogenesis *in vitro*, it was found to be mostly upregulated. Being a glycolytic enzyme, TPI is highly involved in energy metabolism. ATP was shown to be necessary for angiogenic stages, e.g. migration, proliferation and tube formation [21,42,43]. Furthermore, an elevation of TPI expression and of the glycolic metabolism was stated for angiogenic ECs [22]. The angiogenic character of N_1_ might have caused a high expression of TPI from the beginning of cultivation, which was sufficient for cells to migrate and proliferate until day 15. Additional increase of TPI might have facilitated further differentiation of cells. For N_2_, the decrease in TPI mRNA expression at day 15 might have been caused by tip cells being less glycolytically active [21]. HD2 comprises a smaller amount of stalk cells, which might have led to a significantly lower expression of TPI in HD2 in comparison to HD1. In N_1_ and N_2_, VIM mRNA expression decreased in the course of angiogenesis *in vitro*. The highest expression levels were detected in the beginning of cultivation, which most likely represents VIM having its major influence on the cytoskeleton of cells. Therefore, VIM is assumed to have a strong impact on early stages of angiogenesis [15,24,26]. In N_2_, less sprouting and more side-by-side arrangements and networking were visible. This might have demanded a higher activity regarding cell shape and contractility, potentially causing the increase of VIM at day 25. VIM was lately identified as a positive marker for epicardial tip cells [44]. In HD2, a smaller amount of stalk cells were detected, which could have led to VIM being significantly less expressed in HD2 at day 50 compared to HD1. Furthermore, MAT2A mRNA and protein expression fluctuated in both batches during cultivation. First, a decrease was visible. Lately, MAT2A activity was associated with reducing the angiogenic potency of ECs and initiation of cell maturation via SAM [36,37,45]. Therefore, the downregulation of MAT2A in the beginning most likely caused an increase in their angiogenic potency in order to enter first stages of the angiogenic cascade. The following increased mRNA expression, which was also visible on protein level in N_1_, might have initiated cells to enter final stages of angiogenesis. Infection of cells with lentiviral particles appeared to be successful and persistent during the whole cultivation period of 50 days. For SCR_1_, SCR_2_ as well as for sh_1_ and sh_2_, eGFP detection was positive at each day of investigation. No morphological or molecular differences were observed in between control and native groups. By comparing mRNA and protein expression in knockdown groups and control groups, TPI was downregulated in sh_1_ and sh_2_ successfully during the whole experimental period. Comparing TPI mRNA expression between sh_1_ and sh_2_, a higher amount was detected in sh_1_. By them owning more stalk cells, the cell population of sh_1_ was able to produce non-infected cells which might have increased the overall TPI mRNA expression. By excluding the non-infected cells in sh_1_, knockdown groups of both batches displayed a deceleration of *in vitro* angiogenesis by not being able to precede to further stages of angiogenesis *in vitro* than stage 3. As previously described, TPI is highly contributing to cell metabolism of dividing cells [42,46], which might be the reason for knockdown cells not being able to grow towards each other and create a network. Additionally, it has been shown that the sum of assigned stages of knockdown groups ($\bar{S^{{sh}_{1}}},\bar{S^{{sh}_{2}}}$) were significantly smaller than control ($\bar{S^{{SCR}_{1}}},\bar{S^{{SCR}_{2}}}$) and native groups ($\bar{S^{N_{1}}},\bar{S^{N_{2}}}$). Both suggesting that TPI represents a proangiogenic protein which raises the angiogenic potency of ECs *in vitro*.

By knocking down TPI, expressional changes in sh_1_ and sh_2_ considering VIM and MAT2A mRNA were observed. VIM is already described as an angiogenic protein raising the angiogenic potency of HDMECs [15]. With its influence on cell shape and its involvement in Notch ligand signalling, it has a major impact on early stages of angiogenesis, especially during migration and sprouting [24,26,47,48]. Induced by TPI knockdown, cells displayed a decrease in angiogenic potency, which might have led to the downregulation of VIM expression in sh_1_ and sh_2_. In sh_1_, the final uprise in VIM mRNA is most likely caused by non-infected cells amongst the knockdown cells. These ECs were able to enter the angiogenic cascade driven by their unaffected angiogenic potency, resulting in a significant higher VIM expression in comparison to sh_2_. Based on the hypothesis that TPI has a major influence on proliferation of ECs, the knock down might have had a negative influence on their mitogenic activity. As a compensatory mechanism, sh_1_ and sh_2_ might have decreased their MAT2A mRNA expression in the beginning of culture, aiming the reduction of SAM levels. For SAM, a recent study stated its inhibitory influence on growth factors effecting mitosis [45]. For the following upregulation in MAT2A mRNA, both knockdown groups might have adapted to the lower angiogenic activity. MAT2A influences cellular methylation patterns via SAM, which prevents cells to undergo migration and proliferation [36,37].

## Conclusion

This study presents native expression profiles of TPI, VIM and MAT2A during the angiogenic cascade of HDMECs *in vitro*. Indicative of influencing certain stages of angiogenesis *in vitro*, TPI was shown to be strongly expressed throughout angiogenesis, VIM in early stages and MAT2A mostly at the beginning and end. While knocking down TPI, cells were not able to enter late stages of angiogenic cascade *in vitro*, leading to the strong assumption of it being an angiogenic protein having a major impact on cell proliferation. By lowering the angiogenic activity of cells via TPI knockdown, it was stated that the angiogenic protein VIM was downregulated simultaneously. In contrast, MAT2A was mostly upregulated, suggesting its anti-angiogenic influence. Additionally, different batches of HDMECs displayed opposing behaviour after manipulation, despite being from the same distributor and being cultivated under the same conditions. Cell populations with a higher expression of VEGFR-1 and thus a higher amount of stalk cells were able to originate non-infected cells, which were able to enter the angiogenic cascade *in vitro* ending up in late stages. Further investigations are necessary, in order to validate the impact of the three target proteins on *in vitro* angiogenesis and the interaction in between them, e.g. knocking down MAT2A.

## Supporting information

S1 Fig Stages of *in vitro* angiogenesis. The course of angiogenesis is shown for native groups (N_1_, N_2_), control groups (SCR_1_, SCR_2_) and knockdown groups (sh_1_, sh_2_) during a cultivation period of 50 days. Mean values are calculated for 4 visual fields of 4 wells per culture at each detection day. Native and control groups of both batches ran through all six stages of angiogenesis chronologically. Infected cells of sh_1_ and sh_2_ did not precede to further stages than stage 3.
(TIF)

S1 Table Morphologically assigned stages of angiogenesis *in vitro*. Mean values and standard deviations of native, control and knockdown groups of HD1 and HD2 are presented at each day of investigation.
(DOCX)

S2 Table mRNA expression of VIM, TPI and MAT2A. Median and standard error of VIM, TPI and MAT2A mRNA expression of native, control and knockdown groups of HD1 and HD2 are shown at day 5, 15, 25 and 50.
(DOCX)

S3 Table Protein expression of VIM, TPI and MAT2A. Median and standard error of VIM, TPI and MAT2A protein expression of native, control and knockdown groups of HD1 and HD2 are shown in arbitrary Oprical Densitometry units from Western Blot at day 5, 15, 25 and 50.
(DOCX)
