# Supplementary figures and images for: Knockdown of TPI in human dermal microvascular endothelial cells and its impact on angiogenesis in vitro
Source: PLoS One. 2023 Dec 20;18(12):e0294933. doi: 10.1371/journal.pone.0294933 (PMC10732452; doi:10.1371/journal.pone.0294933)

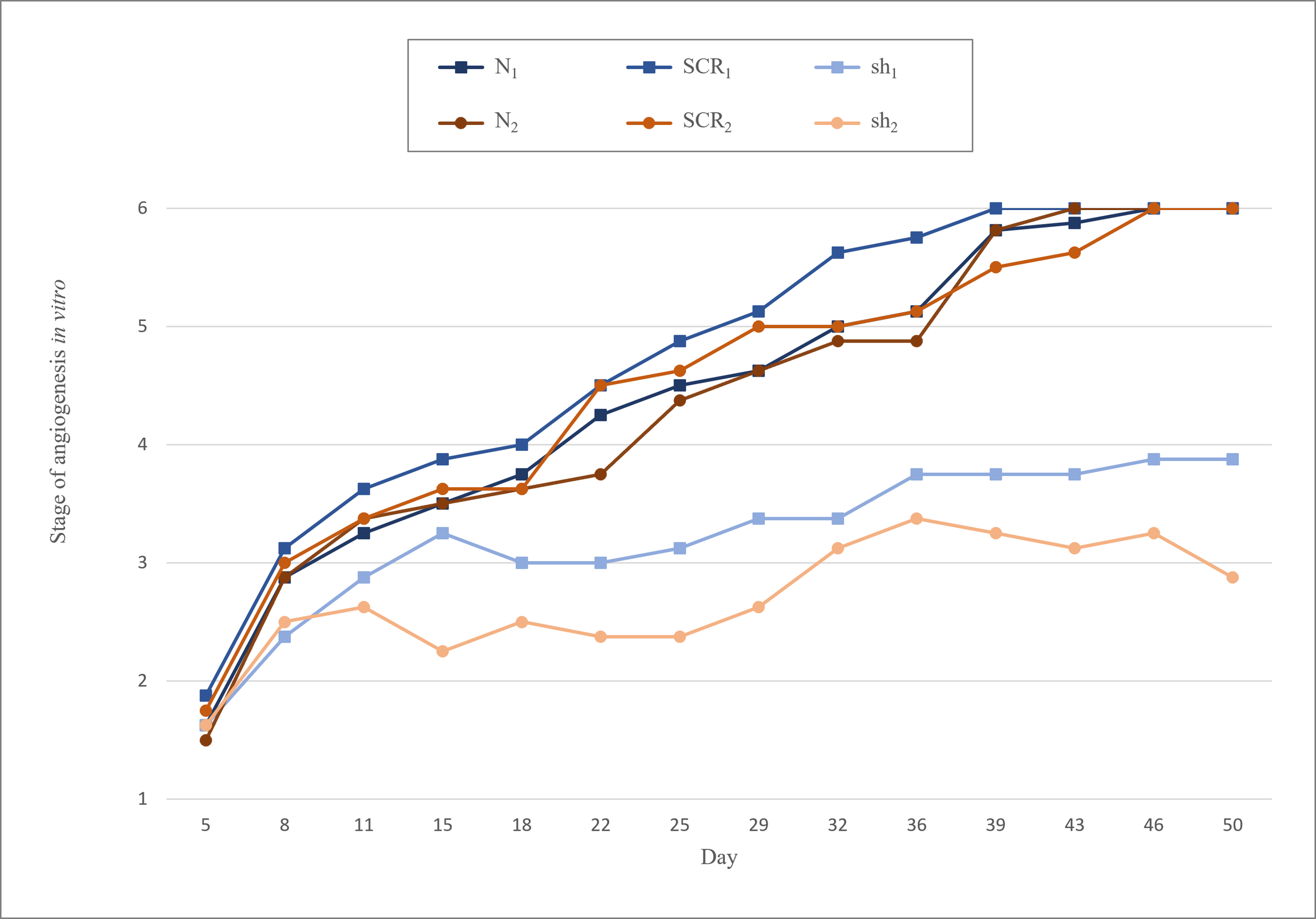

Supplement: S1 Fig — The course of angiogenesis is shown for native groups (N1, N2), control groups (SCR1, SCR2) and knockdown groups (sh1, sh2) during a cultivation period of 50 days. Mean values are calculated for 4 visual fields of 4 wells per culture at each detection day. Native and control groups of both batches ran through all six stages of angiogenesis chronologically. Infected cells of sh1 and sh2 did not precede to further stages than stage 3. (TIF) [file pone.0294933.s001.tif]
