# Supplementary material for: Knockdown of TPI in human dermal microvascular endothelial cells and its impact on angiogenesis in vitro
Source: PLoS One. 2023 Dec 20;18(12):e0294933. doi: 10.1371/journal.pone.0294933 (PMC10732452; doi:10.1371/journal.pone.0294933)
Supplement: S1 Table — Mean values and standard deviations of native, control and knockdown groups of HD1 and HD2 are presented at each day of investigation. (DOCX) [file pone.0294933.s002.docx]

|  | **Day 4** | **Day 8** | **Day 11** | **Day 15** | **Day 18** | **Day 22** | **Day 25** | **Day 29** | **Day 32** | **Day 36** | **Day 39** | **Day 43** | **Day 46** | **Tag50** |
| --- | --- | --- | --- | --- | --- | --- | --- | --- | --- | --- | --- | --- | --- | --- |
| **N_1_** | 1.63 | 2.88 | 3.25 | 3.5 | 3.75 | 4.25 | 4.50 | 4.62 | 5.00 | 5.13 | 5.81 | 6.00 | 6.00 | 6.00 |
|  | ± 0.52 | ± 0.84 | ± 0.71 | ± 0.53 | ± 0.46 | ± 0.71 | ± 0.53 | ± 0.52 | ± 0.53 | ± 0.64 | ± 0.26 | ± 0.35 | ± 0.00 | ± 0.00 |
| **SCR_1_** | 1.88 | 3.13 | 3.63 | 3.88 | 4.00 | 4.50 | 4.88 | 5.13 | 5.63 | 5.75 | 6.00 | 6.00 | 6.00 | 6.00 |
|  | ± 0.64 | ± 0.64 | ± 0.52 | ± 0.35 | ± 0.53 | ± 0.53 | ± 0.35 | ± 0.64 | ± 0.52 | ± 0.46 | ± 0.00 | ± 0.00 | ± 0.00 | ± 0.00 |
| **sh_1_** | 1.63 | 2.38 | 2.88 | 3.25 | 3.00 | 3.00 | 3.13 | 3.38 | 3.38 | 3.75 | 3.75 | 3.75 | 3.88 | 3.88 |
|  | ± 0.52 | ± 0.74 | ± 0.64 | ± 0.71 | ± 0.76 | ± 0.76 | ± 0.83 | ± 0.74 | ± 0.52 | ± 0.71 | ± 0.46 | ± 0.46 | ± 0.35 | ± 0.35 |
| **N_2_** | 1.50 | 2.88 | 3.38 | 3.50 | 3.63 | 3.75 | 4.38 | 4.63 | 4.88 | 4.88 | 5.18 | 6.00 | 6.00 | 6.00 |
|  | ± 0.53 | ± 0.35 | ± 0.91 | ± 0.53 | ± 0.52 | ± 0.46 | ± 0.52 | ± 0.52 | ± 0.35 | ± 0.64 | ± 0.26 | ± 0.00 | ± 0.00 | ± 0.00 |
| **SCR_2_** | 1.75 | 3.00 | 3.38 | 3.63 | 3.63 | 4.50 | 4.63 | 5.00 | 5.00 | 5.13 | 5.50 | 6.00 | 6.00 | 6.00 |
|  | ± 0.46 | ± 0.76 | ± 0.52 | ± 0.52 | ± 0.52 | ± 0.53 | ± 0.52 | ± 0.00 | ± 0.00 | ± 0.35 | ± 0.38 | ± 0.00 | ± 0.00 | ± 0.00 |
| **sh_2_** | 1.62 | 2.5 | 2.63 | 2.25 | 2.50 | 2.38 | 2.38 | 2.63 | 3.13 | 3.38 | 3.25 | 3.12 | 3.25 | 2.88 |
|  | ± 0.52 | ± 0.53 | ± 0.91 | ± 0.46 | ± 0.55 | ± 0.74 | ± 0.74 | ± 0.74 | ± 0.35 | ± 0.52 | ± 0.38 | ± 0.35 | ± 0.89 | ± 0.83 |
