# Supplementary material for: Knockdown of TPI in human dermal microvascular endothelial cells and its impact on angiogenesis in vitro
Source: PLoS One. 2023 Dec 20;18(12):e0294933. doi: 10.1371/journal.pone.0294933 (PMC10732452; doi:10.1371/journal.pone.0294933)
Supplement: S3 Table — Median and standard error of VIM, TPI and MAT2A protein expression of native, control and knockdown groups of HD1 and HD2 are shown in arbitrary Oprical Densitometry units from Western Blot at day 5, 15, 25 and 50. (DOCX) [file pone.0294933.s004.docx]

|  | **Day 5** | **Day 15** | **Day 25** | **Day 50** |
| --- | --- | --- | --- | --- |
| **VIM** | 1142692.24 ± 4989.28 | 1260220.26 ± 3390.15 | 943758.15 ± 5300.33 | 1029055.21 ± 2904.75 |
| **N_1_  TPI** | 851228.18 ± 2453.01 | 496761.81 ± 2029.2 | 792121.16 ± 6276 | 803933.17 ± 8192.5 |
| **MAT2A** | 473978.1 ± 4906.52 | 449147.9 ± 2181.44 | 352864.7 ± 3943.76 | 870074.18 ± 2045.5 |
|  |  |  |  |  |
| **VIM** | 204649.21 ± 7217.85 | 228409.23 ± 2596.18 | 166369.7 ± 1496.92 | 196495.2 ± 4118.56 |
| **SCR_1_  TPI** | 164477.17 ± 4620.5 | 67741.7 ± 1843.71 | 107598.11 ± 6828.03 | 121995.12 ± 6590 |
| **MAT2A** | 85092.8 ± 1218.76 | 66528.69 ± 3605.01 | 66775.06 ± 1978.41 | 149595.15 ± 1307.87 |
|  |  |  |  |  |
| **VIM** | 199272.19 ± 3457.45 | 227384.2 ± 2860.17 | 157590.15 ± 2257.18 | 205004.22 ± 6039.28 |
| **sh_1_  TPI** | 110078.11 ± 4276.05 | 46407.48 ± 1414 | 64720.67 ± 2018 | 107528.42 ± 5154 |
| **MAT2A** | 83029.18 ± 882,97 | 57903.61 ± 4209.1 | 68621.72 ± 4476.12 | 135039.11 ±2100.53 |
|  |  |  |  |  |
| **VIM** | 851289.13 ± 1208.5 | 1250745.62 ± 3110.76 | 934997.19 ± 5516.45 | 1135157.42 ± 5170.87 |
| **N_2_  TPI** | 876875.18 ± 1058.5 | 855424.18 ± 4122 | 959677.2 ± 7282 | 877275.18 ± 3551 |
| **MAT2A** | 607131.3 ± 7271.91 | 730563.15 ± 813 | 503471.01 ± 939.93 | 405217.8 ± 385.5 |
|  |  |  |  |  |
| **VIM** | 119757.6 ± 6822 | 230463.31 ± 1538.25 | 172927.23 ± 5438.69 | 166129.22 ± 1331 |
| **SCR_2_ TPI** | 130982.17 ± 7797.39 | 135644.8 ± 4570.51 | 144528.91 ± 8142.5 | 146368.20 ± 4827 |
| **MAT2A** | 108279.41 ± 6095.91 | 77975.1 ± 3495.5 | 86553.21 ± 2662.17 | 69373.9 ± 4214.1 |
|  |  |  |  |  |
| **VIM** | 127001.17 ± 2463.52 | 224920.3 ± 822 | 182753.52 ± 503.5 | 152240.18 ± 5867.5 |
| **sh_2_  TPI** | 78908.1 ± 5755.98 | 74986.01 ± 3503 | 100268.17 ± 4798.74 | 92180.12 ± 9437.5 |
| **MAT2A** | 103023.2 ± 4841.89 | 85239.11 ± 3504.18 | 100853.2 ± 5985 | 43882.06 ± 397 |
